# The effects of nutrition and health education on the nutritional status of internally displaced schoolchildren in Cameroon: a randomised controlled trial

**DOI:** 10.1017/jns.2024.8

**Published:** 2024-03-13

**Authors:** Mirabelle Boh Nwachan, Richard Aba Ejoh, Ngangmou Thierry Noumo, Clementine Endam Njong

**Affiliations:** The University of Bamenda, Department of Nutrition, Food and Bioresource Technology, Bambili, Cameroon

**Keywords:** Cameroon, Internally Displaced Schoolchildren, Nutrition Education, Nutritional Status, West and Littoral Regions

## Abstract

Lack of nutrition knowledge and poor dietary practices have profound adverse implications on nutritional status particularly among displaced children. Evidence of the effectiveness of nutrition education interventions in improving the nutritional status of internally displaced schoolchildren in Cameroon is scarce. The study objective was to assess the effects of nutrition education on the nutritional status of internally displaced schoolchildren in the West and Littoral Regions of Cameroon. A pre-test-post-test randomised experimental study design was used with an experimental and control group of 160 children from ten primary schools and their caregivers. Anthropometric, biochemical, and clinical signs of malnutrition, dietary, and health status of the children were evaluated, and the caregiver’s nutrition knowledge was assessed in both groups at baseline and end-line with the aid of standard anthropometric and biochemical equipment and recorded in pretested questionnaires. Nutrition education was carried out only with the caregivers of children in the test group. In the test group, significant beneficial outcomes were noticed only for Bitot’s spot (*p* = 0.047), pallor (0.025), frequency of consumption of fruits (*p* = 0.002) and vegetables (*p* = 0.036), caregiver’s nutrition knowledge (*p* = 0.000), all health-seeking practices of the children (*p* < 0.05) except immunisation (*p* = 0.957). No significant change was seen in any of the parameters studied among the participants in the control group. Nutrition education alone was not effective in improving the nutritional status of the children and should be implemented together with other food-based nutrition interventions to improve the nutritional status of internally displaced schoolchildren in the West and Littoral Regions of Cameroon.

## Introduction

Armed conflicts have increased steadily in number, duration, and complexity over the past two decades. Forcible displacement is largely triggered by conflicts, globalisation, and natural catastrophes^(1)^. Disasters caused by natural hazards and complex emergencies such as armed conflicts and civil unrest have resulted in the compulsory displacement of an estimated 108.4 million people in the world and more than 50% of them are internally displaced^(2)^. In Cameroon over 679,393 people were displaced from the Northwest and Southwest Regions as a result of the ongoing Anglophone Crisis and 43% of displaced persons are children^(3)^.

Internally displaced people are among the world’s most vulnerable groups. This is attributed to the fact that conflict-affected people often suffer from the destruction of their agricultural infrastructure, the destruction of health care facilities and convoys that supply humanitarian aid, poor environmental conditions, and poverty. Humanitarian crises result in death, injuries, high prevalence of mental anguish, and outbursts of infectious diseases, principally among internally displaced persons (IDPs) and refugees^(4)^. It significantly augments all forms of malnutrition, illness, and death^(1)^. This is partially because of poor access to nutritious foods since they usually depend on limited food rations and experience physical and financial limitations on access to food markets. The prevalence of acute malnutrition in childhood during emergencies is usually high and may vary from 31% to 80%, with micronutrient deficiencies also more prevalent in IDPs^(2)^. These circumstances are unavoidably associated with increased morbidity and many other socio-economic detriments, which can influence nutritional status^(4,5)^.

Children are an extremely vulnerable group of IDPs. They always suffer more from challenges such as food insecurity, malnutrition, infection, and mortality^(2)^. This is due to their energy and nutrient needs and their vulnerability to infection. Hence, internally displaced children face a disproportionate problem of malnutrition and consequently poor health outcomes.

By virtue of a synergistic relationship with communicable diseases, malnutrition in all its forms has become the leading cause of childhood morbidity and mortality recorded among conflict-affected people^(6)^. Children who are acutely malnourished are by far more likely to become ill and decease. Likewise, children who become ill are more likely to be undernourished. Poor dietary intake is also accountable for the rapid increase of diet-related non-communicable diseases (NCDs), putting an intolerable strain on health systems worldwide^(6)^. Addressing malnutrition among vulnerable populations is of great interest to public health^(7)^.

Under-nutrition is the most prevalent type of malnutrition among conflict-affected children and its consequences extend beyond individuals and families to communities and nations at large. Children suffer the most from the consequences of under-nutrition because they have more nutritional needs to support growth and development. It stunts children’s growth, robs them of fundamental vitamins and minerals, as well as compromises immune function resulting in high morbidity and mortality. Chronic under-nutrition in childhood results in delayed cognitive development with critical long-term health impairments that decrease the quality of life of the individuals concerned^(8,9)^.

Dietary patterns are associated with different short-term complications and major long-term consequences including diabetes, anaemia, CVD, stroke, dental caries, high blood pressure, cancer, asthma, dental caries, and other mental disorders like depression. In addition, adverse outcomes such as low birth weight, disability, poor quality of life, and mortality are also related to poor eating patterns in developing countries^(10)^.

Although school-age is known as a critical stage for shaping eating and lifestyle habits that will last later in life and have an effect during adulthood and even old age^(11)^, the diet of school-aged children in middle- and low-income countries is usually poor in fruits, vegetables, and animal products, leading to inadequate intake of iron and vitamin A. Consequently, school-aged children are at risk of malnutrition associated with inadequate dietary intake^(12,13)^. Thus, addressing the quality and quantity of children’s diets has become a pivotal concern for researchers.

It is well acknowledged that malnutrition during the school years can inhibit a child’s physical and cognitive development resulting in poor academic performance, cognitive achievement, and future earning capacity^(14)^. It is therefore, very imperative to ensure that the nutritional status of schoolchildren is adequate since the foundation of lifetime health and intellectual vigour is laid during this period. Micronutrient deficiencies have life-threatening consequences on schoolchildren’s health. It affects their learning ability, health, and school performance^(11)^. However, attempts to reduce child malnutrition and mortality in developing countries have focused primarily on young children under five years, with little or no effort being directed towards improving the nutritional status of schoolchildren^(15)^.

The prevalence of nutritional deficiencies is high among schoolchildren in developing countries^(16)^. In Cameroon particularly, deficiencies of vitamin A and iron remain a critical public health challenge among children as the prevalence of deficiency of these micronutrients is very high. Vitamin A deficiency (VAD) is 39% and iron deficiency (ID) is 47%^(17)^.

Improving nutritional status and health gives the greatest benefits to the poor and the most vulnerable populations such as internally displaced children. Adequate nutrition, sanitation, and hygiene are essential factors in preventing and treating malnutrition in children affected by emergencies^(18)^. Although many nations have laid down strategies for reducing childhood malnutrition, the question that remains unanswered is whether the Sustainable Development Goals (SDG) of eradicating hunger, achieving food security, and ending all forms of malnutrition by 2030, and promoting sustainable agriculture will be attained in sub-Saharan African, a Region with staggering levels of childhood under-nutrition^(19)^. It was noticed that most of the countries that have had either no progress or insufficient progress in achieving the SDG have experienced a recent crisis or disaster. Since forcibly displaced children are more vulnerable to the consequences of inadequate nutrition, more interventions are needed to ensure adequate energetic and micronutrient intake among them if Cameroon must attain the SDG which are among the central themes of the UN^(7)^.

There is a consensus on specific guidelines for alleviating malnutrition in non-conflict settings such as the promotion of family planning, vitamin A supplementation, vaccination, mass treatment of intestinal parasites, and related programmes. However, specific guidelines on how to tackle malnutrition in conflict settings are still obscure^(20)^. Therefore, more scientific evidence is required in choosing effective nutritional interventions during a humanitarian crisis^(20)^. Although it is well established that food insecurity is one of the major causes of under-nutrition^(1)^, providing food aid to vulnerable persons will only contribute to a short-term solution to malnutrition^(21)^. It is, therefore, indispensable to look for sustainable methods to intensify the fight against malnutrition especially among internally displaced children in Cameroon.

Appropriate evidence-based feeding practices have been proven to be essential for attaining and maintaining a proper nutritional status, and health of displaced children, thus the very survival of these children^(10)^. Although under-nutrition is usually a consequence of either inadequacy of the protective foods necessary for a healthy life, chronic insufficiency of food, or a combination of both situations, it is erroneous to say that the fundamental cause of all nutritional deficiencies is food shortage^(6)^. Even when food is available, less knowledgeable child-caregivers often lack information about appropriate feeding behaviours, which limits their ability to fully utilise the available food resources. According to Chiabi et al.^(22)^, most children in Cameroon suffer from malnutrition not only because of a lack of food but mainly due to poor dietary practices. Deficiency disorders and diseases could also occur as a result of improper food habits, improper child care, abnormal mealtimes, emergencies, poor bioavailability, and food choices^(23)^. Other factors that adversely affect the nutritional status of children are lack of hygiene and other health-seeking practices and prejudices to mention but a few. All these factors can be rectified through a nutrition education intervention^(24)^. Unfortunately, most of the people who are affected by conflicts cannot access the quality nutrition knowledge and care required to prevent or treat malnutrition among their children^(8)^. Hence, the implementation of a nutrition education intervention to address these nutritional challenges is an absolute necessity.

Nutrition education has been implemented among many groups of people to increase awareness about the nutrition problems in the community, to create awareness about the benefits of health-seeking behaviours, and dietary practices, and to improve the knowledge, and decision-making abilities of the target population and consequently their nutritional status. Previous studies have established that knowledge is one of the preliminary steps to changing behaviour. Nutrition knowledge is hence, a fundamental basis for adequate dietary habits. Conversely, insufficient nutrition knowledge is a risk factor for malnutrition^(25–27)^. It has been proven that nutrition education is very effective and has resulted in a momentous improvement in nutrition knowledge and subsequently nutritional status^(24,27,28)^. Also, nutrition education of caregivers/mothers of pre-school aged children in south West Ethiopia effectively improved the anthropometric status of the children^(25)^. In Cameroon, nutrition education of caregivers/mothers in the West Region effectively improved the dietary practices of the children and consequently their nutritional status^(20)^.

However, despite the scientific evidence confirming the positive effects of nutrition education interventions, its effects on the nutritional status of children affected by conflicts and forcible displacement have not been satisfactorily explored in Cameroon^(29)^. Furthermore, to the best of our knowledge, data on the effects of nutrition education on the nutritional status of internally displaced schoolchildren in Cameroon is not available. Therefore, the present study is an effort to design and implement a nutrition education intervention programme aimed at improving the nutritional status of internally displaced schoolchildren by increasing knowledge and behaviour change towards appropriate evidence-based child feeding practices, with particular attention to protein, vitamin A, and iron intake.

## Materials and methods

### Study site

**Fig. 1.**
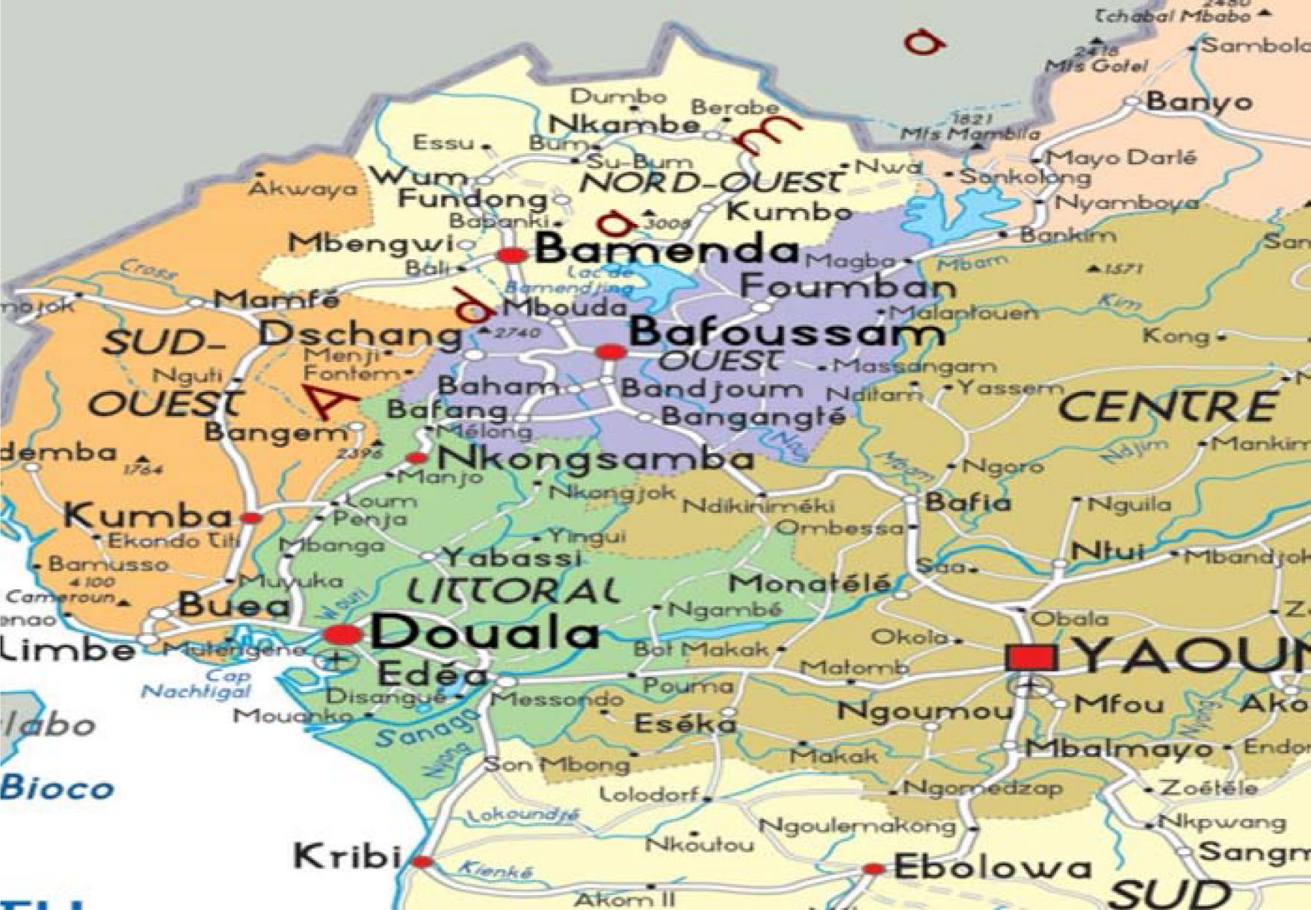
Map showing the West and Littoral Regions.

The study was conducted in the West and Littoral Regions of Cameroon (Fig. 1). Cameroon is a low-to-middle-income, sub-Saharan African country located at the crossroads of West and Central Africa, with a population of over 25 million people. Cameroon is a lower-middle-income country. The country has ten Regions, two of which are occupied by Anglophones^(30)^. Rural areas, which have about 60% of the total population, include 90% of the Cameroonians who live below the poverty line, that is, below 931 FCFA (about 2 USD) per day. Cameroon presently hosts approximately 360,000 refugees from Central African Republic and Nigeria^(30)^.

The West and Littoral Regions share borders with the Northwest and Southwest Regions. Douala which is the economic capital of Cameroon is the Regional capital of the Littoral Region and has a population of more than 2 million people. The Regional capital of the West Region is Bafoussam with a population of 1,843,518 inhabitant and a surface area of 13892 K 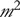 ^(3)^.

### Study design

A pre-test–post-test randomised experimental design which included both an experimental and a control group was used for the study. This study constituted two phases. The first one was a baseline cross-sectional survey conducted to assess the children’s nutritional status and their feeding and health-seeking practices. The mothers/caregivers of the children were taught lessons on nutrition education to improve the nutritional status of the children. During the second phase, an evaluation was done to determine the effectiveness of the nutrition education intervention programme on the nutritional status of the children at the end of the intervention.

### Study population

The study participants were internally displaced schoolchildren (5–15 years) who were displaced by the on-going sociopolitical crisis in the Northwest and Southwest Regions to primary schools of the West and Littoral Regions of Cameroon and their mothers or caregivers.

### Eligibility criteria

#### Inclusion criteria

Pupils who met the following criteria were included in the study: (i) school-aged children within the age range of 5 to 15 years who were internally displaced to the West and Littoral Regions of Cameroon as a result of the ongoing crisis in the North and South West Regions of the country and their caregivers. (ii) Children whose caregivers signed an informed consent for their children to partake in the study. (iii) Children who did not have any chronic illness.

#### Exclusion criteria

Children who did not meet the inclusion criteria above or who were simultaneously participating in any other clinical trial that offered intervention to them or children with any serious illness that needed medical attention were excluded from the study.

### Sample size calculation

The sample size was calculated using the formula for epidemiological studies^(31)^ 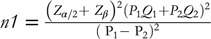


It was calculated based on the following:

*Zα/2* = The critical value, the positive value that is at the vertical boundary for the area in the right tail of the standard normal distribution which is equal to 1.96.

*Zβ* **=** Referred from the tables of normal distribution at the power of 1- *β* where values. *Zβ* is < 0.0 in this case 5% 2-sided test with 80% power *Zβ* = 0.842.

*p1* = the proportion in the target population estimated to have a particular characteristic. The proportion of underweight children between the ages of 5–19 years in Cameroon, which is *p1* is 24%^(7)^.

*Q1* = 1- *p1* expected non-prevalence at the baseline at 76%.

*Q2* = 1- *p2* expected non-prevalence after the interventions at 90%.

*P2* to be 10% was assumed based on an expected 14% reduction rate of the prevalence of underweight among the children. This was substituted in equation *n2* to obtain the number of pupils who participated in the intervention.

Thus, 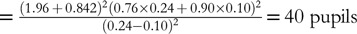


After allowing an attrition rate of 10% (4 pupils), the desired sample was increased to 160 children.

From this formula, a sample size of 40 pupils was obtained and then increased to 160 pupils.

### Sampling technique

The West and the Littoral Regions were purposely selected because these Regions host the majority of IDPs compared to other Regions in the country^(3)^. The Menoua, Bamboutos, and Mifi Divisions in the West Region; as well as Wouri and Moungo Divisions in the Littoral Region were also purposely chosen because these Divisions host the greatest number of displaced children^(3)^. The Divisions in each Region that were used for the experimental and control groups were randomly chosen by balloting. That is, the names of the Divisions were written on separate papers. The papers were folded and mixed and two people who represented either the experimental or control groups were asked to pick the papers. Schools that host large numbers of displaced children were identified by the Regional Delegation of Basic Education during preliminary studies at the Delegation. Ten study schools were randomly selected from this list (six schools from the Littoral Region and four from the West Region). This was done by writing the names of all the schools with large numbers of IDPs on separate papers and folding the papers. Then, someone else was asked to pick six from the Littoral Region and four from the West Region. In the West Region, two randomly selected schools served as experimental schools and two randomly selected schools as control meanwhile in the Littoral Region, three randomly selected schools were used as experimental schools and the same number as control. When selecting study schools, it was ensured that the intervention schools were far from the control schools to avoid the flow of information from those in the intervention group to those in the control group. Depending on the total number of displaced children in the Division, a proportionate number of pupils (boys and girls) were randomly selected from these schools and included in the study. The entire data collection team (except the main investigator who was not involved in assessing the research outcomes) was not informed of the group allocation of the study participants throughout the study period.

### Allocation of group participants and intervention

#### Allocation of group participants

When baseline data collection was finalised, all 160 children in both the test and control groups were dewormed with one tablet of mebendazole 500 mg to prevent parasitic infections from interfering with the effects of the intervention. Then, the 160 children were randomly divided into 2 study groups (either the control or intervention group) of 80 children each along with their mothers/caregivers.

#### Nutrition education intervention

The intervention aimed to improve the nutritional status of the children by increasing the nutrition knowledge of the mothers/caregivers of the children. The nutrition education intervention programme focused on preventing or treating the nutritional problems detected among the children during the baseline survey by imparting good dietary habits in both the children and their mothers/caregivers. They were protein, energy, vitamin A, and iron deficiencies.

Nutrition education was taught to the mothers/caregivers of the children in the intervention group. It was taught by the main investigator in simple vocabulary in pidgin-English or the English language. Each mother/caregiver in the intervention group was taught a lesson on nutrition education weekly. The intervention programme comprised 10 topics and was implemented for 13 weeks. The topics included malnutrition, protein, vitamin A, iron, nutritional deficiency diseases, practices that preserve nutrients in food and avoid wastage of nutrients, balanced diet, planning of low-cost balanced diet for their children, health-seeking practices, and hygiene. Lectures were given to the mothers or caregivers through one-on-one phone calls, text or electronic messages, and booklets with pictures. Only the children in the intervention group were taught hygienic practices such as washing their hands after using the toilet, brushing their teeth every day, and washing fruits before eating. The children and mothers/caregivers in the control group and their parents did not receive any education during the period of the study. At the end of the study, they were given the same booklets that were given to those in the test group.

#### Post-intervention assessment

Post-intervention assessments were done to collect end-line data at the end of the intervention. This was a repetition of all baseline assessments except the socio-demographic assessment. During the end-line assessment, 98.9% of child–mother pairs were assessed and this comprised 80 participants from the intervention group and 78 participants from the control group. The reasons why the children dropped out were because one of them was driven for unpaid school fees and the child failed to return to school after that instance. Also, one child from the control group was transferred from the study school to another school by the parents who had relocated.

### Baseline data collection procedures

The study was done in the West Region from the 13^th^ of September 2021 to the 13^th^ of December 2021 and in the Littoral Region from the 1^st^ of March 2022 to the 30^th^ of May 2022. Baseline and end-line assessments were conducted to assess the impact of the intervention on the expected outcomes. All assessments and data collection were conducted on the school campus. Pre-tested structured interviewer questionnaires were used to collect data at the beginning and the end of the intervention. The questionnaires were administered to the mothers/caregivers of the children in a face-to-face interview during a meeting with them on the school campus. The mothers or caregivers were interviewed either in pidgin-English or in the English language. Information was collected on the demographic information of the children and their mothers/caregivers, drinking water sources, the Region from where the child was displaced, the year of the child’s displacement, dietary and health-seeking practices of the children, morbidity among the children, and the nutrition knowledge of their mothers/caregivers. The following day, the children were measured to collect information on their anthropometry on the school campus, they were also examined for clinical signs of malnutrition, and their blood samples were also collected.

#### Nutritional status assessment

**Anthropometric indicators**

Weight was measured using a digital electronic scale which was calibrated into kilograms and grams (Seca model 750 1017009, China). The weight of the children was measured while wearing light clothes and barefooted with feet apart and looking straight forward. The participants stood still in the middle of the scale’s platform without touching anything and with their weight equally distributed on both feet. The weight was recorded to the nearest 0.01kg. The accuracy of the scale was tested every day before it was used with an object whose weight is known and if it was necessary, the scale was calibrated before use. Height was measured using a portable stadiometer with a movable head piece while children stood erect on bare feet with their heels, buttocks, shoulders, and the back part of the head touching the measuring rod, the hands were freely hanging by the sides. The head was kept comfortably upright with the top of the head having contact with the horizontal head piece. Height was written to the nearest 0.1 cm. A non-stretchable measuring tape calibrated in metres and centimetres was used to measure the mid-upper arm circumference (MUAC) and data was recorded to the nearest 0.1 mm. The measurement was taken on the left arm at the midpoint between the tip of the shoulder and the elbow.

Height, weight, age, and sex were used to compute the height-for-age Z-scores (HAZ), weight-for-age Z-scores (WAZ), BMI-for-age Z-scores (BMIZ), and MUAC-for-age Z-scores (MUACZ) of the children.

**Dietary practices evaluation**

The children’s dietary practices were evaluated using the FFQ and dietary diversity scores (DDSs). The daily meal frequency of the children was also determined.

1. **Meal frequency**

The daily meal frequency of the children was assessed by asking the mothers/caregivers to indicate how many times their children ate meals and snacks in a day.

1. **Dietary diversity**

The method described by Liu et al.^(32)^and Oumer & Berhanu ^(33)^ was used to determine the dietary diversity and micronutrient adequacy of the children at the individual level. This is one of the best methods of assessing the dietary diversity of school-aged children^(34,35)^. The method is based on 10 food groups which are (i) cereals, white roots and tubers, and plantains; (ii) pulses (beans, peas, and lentils); (iii) nuts and seeds; (iv) dairy; (v) meat, poultry, and fish; (vi) eggs; (vii) dark green leafy vegetables; (viii) other vitamin A rich fruits and vegetables; (ix) other vegetables; and (x) other fruits. To remove very small amounts of food, the scoring criterion gives 1 point for a food group when intake is at least 15 g. If not, the score is 0^(36)^. The DDS of an individual was computed by summing the number of food groups eaten by the individual within the previous 24 hours. Minimum dietary diversity (MDD) was defined by a cut-off point of 5 (MDD); that is, children whose DDS was equal to five or more were described as meeting the MDD, and those whose DDS was equal to four or less were defined as not meeting MDD as recommended by FAO^(10)^. Those who ate food from 5 food groups were considered to be of medium dietary diversity, and those from 6 and above were referred to as high diversity score^(10)^

1. **Food frequency determination**

A FFQ was used to assess the usual food selection patterns (variety and frequency) of the displaced children. It was adopted from FAO^(10)^ and customised to incorporate 59 indigenous food items that were found in the local markets or homes of Cameroonians. The questionnaire comprised two sections, which are a list of the food items and a set of frequency-of-use response classifications. The food list is an all-encompassing list of specific food items grouped into subgroups (cereals, roots and tubers, vegetables, legumes and nuts, animal products, fruits, fats, oils, and sugars). Data were collected on the number of times that each child ate food from each food group during the past seven days prior to the study. This includes how often each of the food items included in the FFQ was consumed in a week (less than seven times and seven or more times).

**Blood sample collection procedure and assessment**

Two experienced laboratory technicians were recruited to collect blood samples between 8:00 AM and 10:00 AM to minimise the influence of daily changes on nutrient levels. The samples of blood were collected using standard clinical procedures. The venipuncture was made with a needle to collect blood from the visible vein of the hand. The child’s identification code was used to label the test tubes which contained its blood.

The blood samples were collected before and after the intervention and used to determine the Hb, serum iron, and pre-albumin status of the children. Protein status was analysed through serum pre-albumin concentrations. The reference range for pre-albumin used was 14–26 µg/dL for children 5–12 years and pre-albumin levels of 18–31 µg/dL indicated protein adequacy for children between 13 and 15 years^(37)^. The iron status of the children was assessed by measuring their serum iron concentrations by the Colorimetric method^(38)^. The anaemia status of the children was also assessed by measuring their Hb concentration using the Hb meter (Hemostat GOLD Hemoglobin screening meter, Apex Bio, Taiwan). The cut-off values used to define iron and anaemia status were given by WHO^(39)^.

**Assessment of clinical signs of malnutrition**

Physical examinations were conducted on all children to determine the prevalence of clinical signs of protein-energy malnutrition, vitamin A, and iron deficiencies. The children’s hair, eyes, nails, skin, mouth, legs, and abdomen were examined for clinical signs of protein (depigmentation, bilateral pitting oedema, thin, dry, or sparse hair, distended abdomen, and moon face), vitamin A (night blindness, Bitot’s spots and xerosis of the skin and the conjunctiva) and iron deficiencies (angular cheilitis, easy fatigue, shortness of breath, dizziness, spoon-shaped nails, pallor of the skin, palms, nails, conjunctiva, and tongue). The clinical signs of malnutrition were diagnosed using the symptoms described by Esper^(40)^ and Pogatshnik & Hamilton^(41)^.

**Assessment of morbidity**

The mother/caregiver was asked to recall any form of illness that the index child had experienced within the previous month prior to the study and this information was used to assess the health status of the children. The illnesses that were assessed are those that are associated with childhood protein, vitamin A, and iron deficiencies and included anaemia, dental caries, diarrhoea, vomiting, measles, stomachache, respiratory tract infections (runny nose, cough, wheezing, sneezing, difficulty breathing), malaria, and skin infections. Measles, malaria, and anaemia were only reported if declared by physician diagnosis. Diarrhoea was defined as the sending out of loose or watery feces at least three times a day.

**Health-seeking practices evaluation**

Information on common health-seeking practices of the children such as brushing their teeth daily, washing of hands after using the toilet, washing vegetables before cooking, and washing fruits before consumption, immunisation status and their sources of drinking water was also obtained.

#### Assessment of nutrition knowledge

The mothers/caregivers’ nutrition knowledge was evaluated using a questionnaire that consisted of 10 multiple-choice questions taken from the topics where a knowledge gap was identified during the baseline assessment. At the end of the instructional session, the same questions that were used for pre-intervention assessment to assess nutrition knowledge were given to the mothers/caregivers of the children to determine the change in their nutrition knowledge.

### Statistical analysis

The final data were cleaned, edited, coded, and entered into MS excel sheet. Statistical Package for Social Sciences (SPSS) version^(22)^ was used to analyse the data.

The WHO AnthroPlus Software which has the growth reference for children and adolescents 5–19 years was used to compute HAZ, WAZ, and BMIZ, using the cut-off values given by WHO^(42)^. The reference for MUACZ for school-aged children was used to compute the MUACZ of the children^(43)^. Moderate stunting, moderate wasting, and moderate underweight were defined by HAZ, MUACZ, and WAZ between –3 and –2SD from the median of the WHO reference population, and severe stunting, severe wasting, and severe underweight were defined by HAZ, MUACZ, and WAZ of less than –3. Children whose BMIZ was between < −2.0 and −3.0 SD were considered thin. Children who had BMIZ between −2.0 SD to +1.0 SD and HAZ, MUACZ, and WAZ between −2.0 SD to +2.0 SD were considered normal. Overweight and obesity were defined by BMIZ > +1.0 SD to +2.0 SD and BMIZ > +2.0 SD, respectively^(42)^.

The mothers/caregiver’s responses to questions on nutrition knowledge were marked as zero mark for a wrong response, and one mark for a correct response. The questionnaires were marked on 10. The mothers who scored less than 3 marks out of 10 were said to have very poor nutritional knowledge. Mothers whose marks were 3 or 4 out of 10 were said to have poor nutrition knowledge. Those who had 5 or 6 out of 10 were said to have averagely good nutrition knowledge, and a mark of 7 or 8 out of 10 was defined as very good nutrition knowledge while a mark of 9 or 10 out of 10 was defined as excellent nutrition knowledge.

A one-way ANOVA and Pearson’s chi-square test were used to test for significant differences where appropriate and the significance level was set at *p* < 0.05 with 95% confidence limit.

The results were presented using tables, figures, and charts such as bar charts, pie charts, and line graphs. Continuous data were presented as median with range or mean ± SD, while categorical data were presented as frequencies, proportions, means, and correlation coefficients were computed. A *p* ≤ 0.05 was considered statistically significant.

### Informed consent and ethical approval

The study was conducted according to the ethical guidelines of the 2008 Helsinki Declaration. Ethical approval for the study was obtained from the institutional ethics committee and the institutional ethics committee of the University of Bamenda (Ref No: 2021/006H/UBa/IRB). The Regional Delegate of Basic Education for the West and Littoral Regions gave the authorisation to carry out the study in their respective Regions. Also, the Divisional delegates, Inspectorates of Basic Education, and Head teachers of the selected schools gave additional administrative approvals for the study. Each mother/caregiver signed an informed consent for their children to participate in the study. Before the informed consent was signed, the aim and study method were explained during a meeting held on the school campus with the mothers/caregivers of the children.

## Results

### Socio-demographic characteristics of the children and mothers/caregivers

#### Demographic characteristics of the children

The experimental and control groups were alike at baseline in terms of socio-demographic data and no significant difference was found between the groups at baseline. The demographic characteristics of the children at baseline are presented in Table 1. The results showed that there were slightly more females in both the experimental and control groups (53.8% and 56.3% respectively) than males. The children in the intervention group were marginally older than those in the control group as 58.7% of those in the intervention group were between 10 and 15 years meanwhile only 57.5% of children in the control group were in this age range. Most of the children in both the intervention group (72.5%) and the control group (76.3%) were displaced from the Northwest Region. The intervention and control groups were comparable at baseline in terms of mean height (126.1 ± 4.21cm for the intervention group, and 125.57 ± 5.22 cm for the control group), mean weight (28.47 ± 2.35 kg for the intervention group, and 28.36 ± 2.41kg for the control group), mean MUAC (181.5 ± 5.25 cm for the intervention group, and 172.8 ± 6.37cm for the control group). The highest number of children were displaced in 2018 as the highest proportion of children in both the intervention group (37.5%) and the control group (35%) were displaced in that year. Nearly half of the children in both the intervention group (45%) and the control group (37.5%) lived in crowded houses with more than eight people per house.

**Table 1.** Demographic characteristics of the children at baseline

Child’s characteristics	Category	Experimental	Control
		Frequency (%)	Frequency (%)
Sex of child	Male	37 (46.2)	35 (43.8)
	Female	43 (53.8)	45 (56.3)
Age range of children	5–9 years	33 (41.3)	34 (42.5)
	10–15 years	47 (58.7)	46 (57.5)
Height (cm)	Mean	126.1 ± 4.21	125.57 ± 5.22
Weight (kg)	Mean	28.47 ± 2.35	28.36 ± 2.41
MUAC (mm)	Mean	181.5 ± 5.25	172.8 ± 6.37
Region where the child lived before the crisis.	Northwest	58 (72.5)	61 (76.3)
	Southwest	22 (27.5)	19 (23.8)
The year of child displacement.	2017	16 (20)	18 (22.5)
	2018	30 (37.5)	28 (35)
	2019	26 (32.5)	15 (18.8)
	2020	7 (8.8)	11 (13.8)
Household size.	2021	1 (1.3)	8 (10)
	Below 5	9 (11.3)	6 (7.5)
	5–8	35 (43.8)	44 (55)
	More than 8	36 (45)	30 (37.5)

#### Demographic characteristics of the mothers/caregivers

At baseline, the intervention and the control groups were analogous in terms of marital status (65% married in the test group, 68.7% married in the control group) as presented in Table 2. The rate of unemployment was high among the mothers/caregivers of the children in both groups (> 40%). Generally, the families of the displaced children had a low income. At least 60% of the families in both groups had a monthly income of less than 50,000 Francs CFA (< 100 USD). The primary and secondary levels of education were the most common highest levels of education of the mothers/caregivers of the children in both the experimental (37.5%, and 52.5 respectively) and control (25%, and 70% respectively) groups. Some of the children (11% and 16 % in the test and control groups) drank water from bad sources such as streams, and wells, and more than three-quarters of them used the pit toilet (test group = 87.6%; control group = 77.5%). Most of the mothers/caregivers were Christians (test group = 86.3%; control group = 80%).

**Table 2.** Socio-demographic data of the mothers/caregivers at baseline

Mother’s/caregivers’ characteristics	Category	Experimental	Control
		Frequency (%)	Frequency (%)
	No formal education	2 (2.5)	2 (2.5)
The highest academic level of mother/caregiver	Primary	30 (37.5)	20 (25)
	Secondary	42 (52.5)	56 (70)
	Higher	6 (7.5)	2 (2.5)
Employment status of the mother /caregiver	Formally employed	3 (3.7)	11 (13.8)
	Self-employed	28 (35)	37 (46.3)
	Unemployed	49 (61.3)	32 (40)
	Married	52 (65)	55 (68.7)
Marital status of mother/caregiver	Divorced	4 (5)	3 (3.8)
	Never married	16 (20)	19 (23.8)
	Widow	8 (10)	3 (3.8)
	Below 50,000	67 (83.8)	48 (60)
	50,000–150,000	12 (15)	27 (33.8)
Household income	151,000–300,000	0 (0)	5 (6.3)
	Above 300,000	1 (1.3)	0 (0)
	Mother	45 (56.3)	48 (60)
Relationship of mother/caregiver to child	Family relation	35 (43.7)	32 (40)
	Parent’s friend	0 (0)	0 (0)
	Others	0 (0)	0 (0)
	Below 25	38 (47.5)	27 (33.8)
Age of mother/caregiver	25–50	37 (46.3)	47 (58.8)
	Above 50	5 (6.3)	6 (7.5)
	Renting	69 (86.3)	54 (67.5)
Housing condition	Living under another family	11 (13.8)	21 (26.3)
	House owner	0 (0)	5 (6.3)
Main drinking water source	Good water source	67 (83.8)	71 (88.8)
	Bad water source	13 (16.2)	9 (11.2)
Toilet type	Pit Toilet	70 (87.6)	62 (77.5)
	Flushing Toilet	10 (1.3)	17 (21.3)
	No Toilet	0 (0)	1 (1.3)
Religion	Christian	61 (86.3)	64 (80)
	Muslim	19 (23.8)	14 (17.5)
	Pagan	0 (0)	2 (2.5)
	Others	0 (0)	0 (0)

### Impact of nutrition education and counselling on the anthropometric status of the displaced schoolchildren

As shown in Table 3, there was a general increase in the weight and height of all the children in both groups but the increase observed in the experimental group (0.37 kg) was slightly more than what was observed in the control group (0.35 kg).

**Table 3.** Mean Z-score of the children before and after the nutrition education intervention

Intervention group					
	Baseline	End-line			
Variable	Mean (SD)	Mean (SD)	F-stat (df)	*p*-Value	Effect size
Weight (kg)	26.68 (±2.75)	27.05 (±2.62)	1.62 (1, 156)	0.177	0.072
Height (cm)	120.7 (±9.12)	123.1 (±9.47)	0.94 (1, 156)	0.135	0.108
BMIZ	–1.58 (±0.13)	–1.54 (±0.18)	0.51 (1, 156)	0.104	0.031
WAZ	–1.64 (±0.33)	–1.61 (±0.32)	0.34 (1, 156)	0.571	0.063
HAZ	–1.69 (±0.37)	–1.68 (±0.34)	0.16 (1, 156)	0.790	0.017
MUACZ	–1.56 (±0.34)	–1.49 (±0.25)	2.68 (1, 156)	0.070	0.044
Control group					
Weight (kg)	26.91 (±2.50)	27.26 (±2.54)	0.76 (1, 156)	0.151	0.027
Height (cm)	122.2 (±8.34)	124.5 (±7.26)	0.63 (1, 156)	0.984	0.041
BMIZ	–1.54 (±0.28)	–1.56 (±0.12)	0.80 (1, 156)	0.581	0.019
WAZ	–1.57 (±0.33)	–1.62 (±0.24)	1.04 (1, 156)	0.197	0.021
HAZ	–1.71 (±0.51)	–1.73 (±0.41)	0.31 (1, 156)	0.713	0.035
MUACZ	–1.59 (±0.36)	–1.62 (±0.28)	0.42 (1, 156)	0.486	0.046

At baseline, the mean Z-scores of BMI-for-age, WAZ, HAZ, and MUAC-for-age were –1.54 ± 0.28, –1.67 ± 0.33, –1.71 ± 0.51, and –1.59 ± 0.36 in the control group and –1.58 ± 0.23, –1.64 ± 0.33, –1.69 ± 0.37, and –1.56 ± 0.34 in the experimental group respectively.

At the end of the study, an ANOVA revealed that there was no significant (*p* > 0.05) improvement in the WAZ, HAZ, BMIZ, and MUACZ of the children in both the control and the experimental groups and the effect size was small (< 0.06) for all the parameters studied except weight in the experimental group which was medium (0.072).

### Impact of nutrition education on the biochemical status of children

During the baseline data collection, it was noticed that the mean serum pre-albumin, Hb, and serum iron of the children in the intervention group was 18.7 ± µg/dL g/L, 11.8g ± 2/dL, and 93.6 ± 10 mcg/dL respectively meanwhile for the control group, it was 39.3 ± 3g/L, 12.0g ± 3/dL, and 94.8 ± 12 µ/dL respectively. At post-intervention assessment, an ANOVA showed that the effect of nutrition education on the mean of serum pre-albumin, Hb, and serum iron of the children in the intervention group was insignificant (*p* > 0.05) as shown in Table 4. Also, the effect size was small (< 0.06) for all the parameters studied confirming that these differences are practically insignificant.

**Table 4.** Effects of nutrition education on biochemical parameters

Experimental group					
	Baseline	End-line			
Variable	Mean (SD)	Mean (SD)	*F*-stat (df)	*p*-Value	Effect size
Serum pre-albumin (µg/dL)	18.5 ± 0.6	18.7 ± 0.4	0.23 (1, 156)	0.794	0.052
Hb (g/dl)	11.8 ± 2	12.3 ± 3	0.91 (1, 156)	0.166	0.030
Serum iron (µ/dL)	93.6 ± 10	96.1 ± 9	1.30 (1, 156)	0.079	0.017
Control group					
Serum pre-albumin (µg/dL)	18.7 ± 0.3	18.8 ± 0.7	0.47 (1, 156)	0.945	0.023
Hb (g/dL)	12.0 ± 3	11.8 ± 4	0.32 (1, 156)	0.716	0.046
Serum iron (µ/dL)	94.8 ± 12	93.6 ± 13	0.45 (1, 156)	0.588	0.018

### Impact of nutrition education and counselling on clinical signs of malnutrition

In the intervention group, some of the clinical signs of malnutrition such as depigmentation, thin, dry, or sparse hair, moon face, and Bitot’s spot noticed among the children at baseline were normalised at the end-line, although only Bitot’s spots and pallor had significant changes (*p* < 0.05) (Table 5). No significant change was observed in the control group (*p* > 0.05).

**Table 5.** Impact of nutrition education on clinical signs of malnutrition

	Experimental group				Control group		
Type of deficiency	Clinical sign	Baseline *n* (%)	End-line *n* (%)	*p*-Value	Baseline *n* (%)	End-line *n* (%)	*p*-Value
Protein	Depigmentation	5 (6.3)	2 (2.5)	0.242	3 (3.8)	4 (5.1)	0.691
	Thin, dry, or sparse hair	4 (5.0)	1 (3.3)	0.548	5 (6.3)	5 (6.4)	0.931
	Moon face	2 (2.5)	1 (1.3)	0.317	1 (1.3)	1 (1.3)	0.865
Iron	Pallor	8 (10.0)	2 (2.5)	0.025	6 (7.5)	8 (10.2)	0.551
	Dizziness	1 (1.3)	0 (0)	0.314	0 (0)	1 (1.3)	0.307
	Angular cheilitis	2 (2.5)	1 (1.3)	0.579	2 (2.5)	3 (3.8)	0.640
Vitamin A	Xerosis	5 (6.3)	3 (3.8)	0.471	6 (7.5)	5 (6.4)	0.914
	Bitot’s spot	2 (2.5)	0 (0)	0.047	0 (0)	0 (0)	1
	Night blindness	0 (0)	0 (0)	1	1 (1.3)	1 (1.3)	1

### Effects on dietary practices

#### Impact of the intervention on number of meals per day

According to Table 6, most of the children in the control group (68%) consumed two meals per day in comparison with the experimental group where only 48.8% of the children consumed meals thrice daily at baseline. At the end of the intervention, there was no significant improvement in the daily meal frequency of the children in both the experimental and control groups.

**Table 6.** Impact of nutrition education on the daily meal frequency of children

	Experimental group			Control group		
No. of meals per day	Baseline *n* (%)	End-line *n* (%)	*p*-Value	Baseline *n* (%)	End-line *n* (%)	*p*-Value
One	2 (2.5)	2 (2.5)	1	0 (0)	0 (0)	1
Two	37 (46.3)	32 (40)	0.422	44 (55)	53 (68.0)	0.074
Three	41 (51.3)	45 (56.3)	0.527	31 (38.7)	22 (28.2)	0.198
> Three	0 (0)	1 (1.3)	0.307	5 (6.3)	3 (3.8)	0.583

#### Impact of nutrition education on the dietary diversity scores of children

**Fig. 2.**
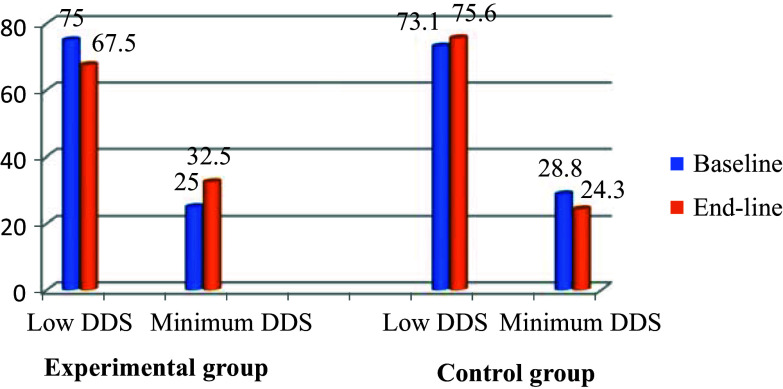
Impact of nutrition education on the dietary diversity scores of children.

The mean DDS of the children in the experimental group at baseline was slightly higher (2.911 ± 0.13) than the baseline mean DDS of children in the control group was 2.85 ± 0.25 (Fig. 2). At the end-line, no significant changes were reported in the DDS of the children in both the experimental and the control groups. The percentage of children with medium DD at baseline was 17.9% and 20% in the experimental and control groups respectively. Non-significant improvements were observed in the DDSs of the children of the intervention group after the nutrition education intervention.

#### Impact of nutrition education on the weekly frequency of food intake of children

At baseline, there were no significant differences in the frequency of foods selected by the mothers/caregivers between the two groups. The results in Table 7 indicate that both groups ate predominantly a carbohydrate-rich diet (cereals) both before and after the nutrition education intervention, with minimal animal protein, fruits, or vegetables. During the post-intervention assessment, it was noticed that only the frequency of consumption of fruits and vegetables increased significantly. No significant change was reported in the frequency of consumption of all the other food groups. The proportion of children who consumed cereals, legumes and nuts, roots and tubers, fruits, animal products, and vegetables weekly in the control group did not change statistically after the intervention.

**Table 7.** Impact of nutrition education on the weekly frequency of food intake of children

	Baseline (%)	End-line (%)	*p*-Value
Experimental group	≥7 times	≥times	
Cereals	93.8	87.5	0.174
Vegetables	52.5	68.75	0.036
Fruits	47.5	71.3	0.002
Animal products	31.3	33.8	0.738
Roots and tubers	33.8	35	0.874
Legumes and nuts	36.3	40	0.633
Fats, oils, and sugar	100	100	1
Control group			
Cereals	95	97.4	0.432
Vegetables	50	47.4	0.744
Fruits	46.3	43.8	0.752
Animal products	28.8	24.4	0.532
Roots and tubers	28.8	29.5	0.923
Legumes and nuts	26.3	23.1	0.642
Fats, oils, and sugar	100	100	1

### Impact of the intervention on maternal nutrition knowledge

#### Impact of the intervention on maternal mean nutrition knowledge score

**Fig. 3.**
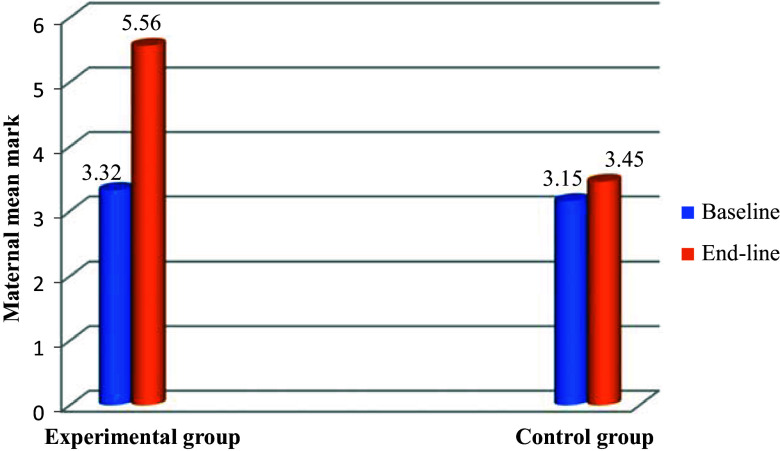
Impact of intervention on maternal mean knowledge score.

The mean baseline nutrition knowledge score was 3.15 ± 0.13 in the control group and 3.32 ± 0.22 in the intervention group. The mean nutrition knowledge score of subjects in the intervention and control groups at the end-line was 5.56 ± 0.35and 3.45 ± 0.17, respectively. At the end of the intervention mothers in the nutrition education intervention group scored significantly higher (*p* = 0.000) meanwhile no significant change was observed in the control group as shown in Fig. 3 (*p* = 0.102).

#### Effect of intervention on various levels of nutrition knowledge

At baseline, the level of nutrition knowledge of most of the mothers/caregivers in both the control (63.8%) and the experimental (60%) groups was poor and without any significant differences between the groups as shown in Table 8. At baseline, the percentage of mothers with very poor knowledge, poor knowledge, average knowledge, very good knowledge, and excellent knowledge in the experimental group was 22.5%, 37.5%, 28.8%, 8.7%, and 2.5%, respectively and these percentages improved (*p* < 0.05) respectively to 7.5%, 17.4%, 43.8%, 25% and 6.3% at end-whereas, in the control group it remained at 23.8%, 40%, 26.3%, 9.9%, and 0%, respectively.

**Table 8.** Effect of intervention on various levels of nutrition knowledge

	Intervention group			Control group		
Level of nutrition knowledge	Before *n* (%)	After *n* (%)	*p*-Value	Before *n* (%)	After *n* (%)	*p*-Value
Very poor knowledge	18 (22.5)	6 (7.5)	0.007	19 (23.8)	18 (23.8)	0.905
Poor knowledge Average knowledge	30 (37.5) 23 (28.8)	14 (17.4) 35 (43.8)	0.004 0.049	32 (40) 21 (26.3)	32 (41.3) 20 (25)	0.764 0.682
Very good knowledge	7 (8.7)	20 (25)	0.007	8 (9.9)	8 (9.9)	0.939
Excellent knowledge	2 (2.5)	5 (6.3)	0.237	0 (0)	0 (0)	1

The results of the study showed a significant increase in all the levels of nutrition knowledge for the mothers/caregivers in the experimental group after receiving the nutrition education, while the average score of the mothers/caregivers in the control group did not change over the same time frame.

### Impact of nutrition education on the health status of the children

From the results in Table 9, many illnesses were reported among the children in both groups at baseline and end-line but the most common were respiratory infections such as catarrh and cough. No significant improvement was reported in the prevalence of illnesses among the children in the experimental group. Likewise, no significant change was observed in the prevalence of the illnesses among the children in the control group.

**Table 9.** Impact of nutrition education on the health status of the children

Morbidity	Intervention group			Control group		
	Before *n* (%)	After *n* (%)	*p*-Value	Before *n* (%)	After *n* (%)	*p*-Value
Dental caries	10 (12.5)	11 (13.8)	0.905	5 (6.3)	5 (6.4)	0.903
Diarrhoea Vomiting	12 (15) 2 (2.5)	8 (10) 1 (1.3)	0.548 0.361	3 (3.8) 0 (0)	2 (2.6) 0 (0)	0.669 1
Stomachache	13 (16.3)	10 (12.5)	0.498	9 (11.3)	9 (11.5)	0.968
Respiratory infections	22 (27.5)	23 (28.8)	0.389	18 (22.5)	20 (25.6)	0.649
Malaria	3 (3.8)	0 (0)	0.302	4 (5)	2 (2.6)	0.432
Skin infections	8 (10)	7 (8.987)	0.876	6 (7.5)	6 (7.7)	0.962

### Impact of nutrition education on health-promoting practices

At baseline, the health-seeking practices of the children in both groups were sub-optimal as not up to half of them had any good health-seeking practices as presented (Table 10). Nutrition and health education significantly improved all the health-promoting practices of the children except immunisation. No significant change was observed in the control group after intervention.

**Table 10.** Impact of nutrition education on health-promoting practices

Health-seeking practices	Intervention group			Control group		
	Before *n* (%)	After *n* (%)	*p*-Value	Before *n* (%)	After n (%)	*p*-Value
Complete immunisation	78 (97.5)	78 (97.5)	0.957	80 (6.3)	78 (6.3)	0.993
Brushed teeth daily Washed fruits before eating	28 (35) 32 (40)	55 (68.8) 45 (56.3)	0.000 0.039	25 (31.3) 29 (36.3)	27 (34.6) 33 (42.3)	0.660 0.441
Washed hands after using the toilet	27 (33.75)	40 (50)	0.037	30 (37.5)	28 (35.9)	0.835

## Discussion

The prevalence of poverty among most of the mothers/caregivers (≥ 60%) was high as indicated by very low household income levels of less than 50.000Francs CFA per month. This probably affected negatively the nutritional status of the children and the effects of the intervention.

At the end-line, it was noticed that nutrition education did not result in improvement in HAZ, WAZ, MUACZ, and BMIZ of the children. The lack of significant improvement in HAZ, WAZ, and MUACZ after the intervention is consistent with the results of Mushaphi et al.^(44)^ who did not observe any significant improvement in the nutritional status of young children in Limpopo Province, South Africa after a nutrition education intervention of the mothers/caregivers. The present finding is also comparable with previous studies conducted by Mananga et al.^(27)^ in rural Cameroon and also with another study in Somalia which provided nutrition education to mothers of internally displaced children who were living in Somalian camps^(45)^. They did not notice any statistically significant difference in underweight, and wasting among the study children after nutrition education of their mothers. Other studies on the impact of nutrition education and counselling on the nutritional status of adolescents reported the same results among children in the Ashanti Region of Ghana, where a positive but insignificant improvement was observed in the children’s nutritional status at the end of a nutrition education intervention^(46)^.

The overall absence of a significant reduction in stunting, wasting, and underweight prevalence in this study was an unexpected result but could be justified by the problems associated with the deplorable living conditions of IDPs such as over-crowded houses, morbidity, lack of sanitation, inaccessibility to markets and health care services, deterioration of mental health, and food shortages which cannot be solved by nutrition education and counselling only. A possible explanation for the absence of outstanding improvement in the indicators of the children’s nutritional status observed in this study could also be that malnutrition has been proven to be multifactorial, and no single intervention implemented alone is likely to be appropriate to maintain a significant decrease in under-nutrition^(46)^. The poor nutritional status of the displaced children could be caused by lack of access to nutritious food and insufficient nutrition knowledge, and this may account for the lack of success of this intervention. This suggestion was affirmed by a scoping review among internally displaced children in sub-Saharan Africa which also revealed that failure of nutritional interventions for internally displaced children could also be a consequence of a lack of access to nutrient-rich food rather than inadequate knowledge of healthy dietary practices^(47)^. Again, a study in Uganda reported that lack of material, financial, and human resources were among the major limitations of a nutritional intervention among conflict-affected schoolchildren in northern Uganda^(48)^. Hence, for internally displaced children, interventions that are only nutrition education-based might not be sufficiently effective. Thus, food-based nutrition interventions such as humanitarian aid to increase food access among displaced children in the West and Littoral Regions should be intensified alongside the provision of nutrition education to make the intervention more effective.

Another justification for the absence of improvement may also be because most of the children had a normal anthropometric status at baseline, leaving only small room for improvement. The fairly short period of intervention might also not have given sufficient time for significant physiological modification in nutritional status to take place. Consequently, the results of the present study suggest that more qualitative research should be done to unravel the contextual factors associated with the success of intervention studies and to identify the practices that will help enhance the nutritional status of internally displaced children.

Nutrition education has been proven to be one of the most sustainable and cost-effective strategies for preventing and treating macronutrient and micronutrient malnutrition in non-conflict settings^(14)^. Although the present study was not carried out in conflict settings, it was conducted among a conflict-affected population and this might be the reason why nutrition education failed to improve the nutritional status of the children. This observation is contrary to the study of Salehi et al.^(28)^ who reported that nutrition education significantly reduced all forms of malnutrition among Iranian nomadic children whose mothers participated in a nutrition education programme for 12 months. The results of the current study are also dissimilar to the result of a systematic review conducted on the impact of nutrition education on the nutritional status of young children in less developed countries^(24)^. They reported a significant decrease in wasting among the children included in the study^(24)^. Nutrition education was also effective in preventing and treating under-nutrition and micronutrient deficiency among young children in western Uganda and primary schoolchildren in Malaysia^(49,50)^ The difference in findings noticed between the current study and these previous studies may be due to the lack of intervention for other factors and confounders in the present study. It may also be because the children in the above-mentioned studies were not forcibly displaced. This is because a scoping review that focused on the efficacy of nutrition education in improving the nutritional status of displaced children in the sub-Saharan African Region demonstrated that nutrition education interventions solely could not improve the nutritional status of forcibly displaced children. Rather, humanitarian aid agencies and the ability of the displaced families to relate with their host communities improved the nutritional status of the children^(47)^.

There were no significant improvements in stunting in both the test and control groups at the end-line. However, the HAZ of children in the intervention group improved slightly meanwhile the children in the control group experienced some growth faltering at the end-line, as indicated by their HAZ. These findings demonstrate that the nutritional status of internally displaced children in the West and Littoral Regions of Cameroon might keep deteriorating if no additional intervention is put in place to curb malnutrition among the displaced children. Therefore, interventions to curb malnutrition should be implemented among all the displaced children in these Regions.

The change in the mean HAZ of stunting was insignificant in both groups from baseline to end-line. Indeed, stunting is a consequence of prolonged episodes of insufficient intake of food and augmented morbidity, and it commonly occurs in children living in areas of poor socio-economic settings. It is associated with poor cognitive development in children less than five years old. Hence it is likely that most of the diets given to the children were insufficient to satisfy their physiological requirements for growth. The nutrition education intervention did not have a significant effect on stunting possibly owing to its short duration (13 weeks) and the fact that food supplementation was not included.

The insignificant reduction in stunting observed among children whose mothers were taught adequate dietary practices is consistent with previous studies conducted among children in Limpopo Province, South Africa^(44)^ and in rural Cameroon^(27)^. Moreover, nutrition counselling for mothers of internally displaced children living in camps in Somalia did not affect stunting among their children^(45)^. All these authors established an insignificant reduction in stunting among children after their mothers/caregivers were educated with nutrition counselling^(27,44,45)^.

The baseline prevalence of protein, iron, and Hb deficiencies noticed among the children in this study was high. This may be attributed to the fact that the diet of most of the displaced children consists largely of plant-based foods which generally are low in essential nutrients like iron and protein^(12)^. Rather their food contains anti-nutrients which constrain the bioavailability of some micronutrients. This demonstrates the need for nutritional interventions that include the provision of nutritious foods that are rich in these nutrients to the children.

The end-line results of this study reveal that the serum iron and Hb levels of the children did not increase significantly. This might be due to the high prevalence of morbidity, chronic under-nutrition, and multiple micronutrient deficiencies among the study children at baseline. These results corroborate earlier studies in Limpopo Province, South Africa where 12 months of nutrition education for mothers/caregivers insignificantly improved the Hb and iron status of their children^(44)^. Furthermore, a nutritional intervention that involved nutrition education of the children’s caregivers had no statistically significant effect on the Hb status of undernourished children in Aceh, Indonesia^(51)^.

Most behavioural interventions that are food-based always focus on increasing the intake of single foods or single nutrients^(52,53)^. However, the strength of the current intervention is that it encouraged the consumption of a variety of foods and this helped the mothers/caregivers to significantly increase their children’s frequency of intake of fruits and vegetables. It is a well-known fact that the intake of a wide variety of foods is related to elevated consumption of micronutrients^(10)^. The benefits of the mutual effects of increased nutrient intake and hygiene could account for the improvement witnessed in the clinical signs of malnutrition among the children in the test group at post-intervention.

Although the nutrition education intervention resulted in significant improvement in the frequency of consumption of fruits and vegetables among the children, it did not result in significant improvement in their iron and Hb status. This is probably because their food sources were mostly plant-based^(12)^. Hence, the anti-nutrient substances in plant-based foods hinder the bioavailability of iron, resulting in an insignificant increase observed after the intervention. Furthermore, the intake of vegetables and fruits was low among the children in all the groups both at baseline and end-line. This means that the intake of vitamin C among children is low and vitamin C improves the absorption of iron from the diet.

Contrarily, the Hb and iron status of children suffering from ID anaemia in rural Cameroon improved significantly after their mothers were taught nutrition education lessons^(27)^. One of the most obvious reasons for the remarkable improvement in the study by Mananga et al.^(27)^ was the provision of iron supplements for 16 weeks. The substantial improvement could also be attributed to the iron and anaemia status of the children before the intervention. The selected children were either iron deficient or anaemic, and this leaves much room for improvement to take place during an intervention, unlike the case of the internally displaced children in the present study who were mostly normal.

Nutrition education significantly reduced the prevalence of some clinical signs of malnutrition such as pallor and Bitot’s spots. The reason why nutrition education had no significant effect on the anthropometric status of the children but on clinical signs could be because clinical signs of malnutrition are visible and hence the caregivers of the concerned children took the nutritional counselling more seriously and acted upon it accordingly, giving special treatment to the children concerned meanwhile the other caregivers considered their children to be normal and therefore did not go any extra miles to implement the teachings they received because of shortage of the required resources.

An insignificant improvement was observed in the prevalence of most of the clinical signs of malnutrition studied among the children such as depigmentation, thin, dry, or sparse hair, and moon face. One of the reasons for the insignificant improvement could be that some of the signs seen may be a result of a non-nutritional condition such as harsh weather, poor hygiene, or excessive exposure to the sun since clinical signs are disreputably non-specific. Again, clinical signs are mostly visible at the existence of the disease and may not correlate with dietary intake data or the biochemical values in the individual or the population. Although they may be the first warning sign that something is amiss, they are usually only the first step in a complex chain of evidence, which requires other investigations, before a diagnosis can be made with confidence^(54)^. The negligible effect of the nutrition education intervention programme on clinical signs of malnutrition necessitates additional studies of underlying causes of malnutrition among children.

Since nutrition education had a significant effect on some clinical signs of malnutrition but had an insignificant effect on other forms of malnutrition such as wasting and being underweight, it can be inferred that nutrition education is an effective strategy in reducing the prevalence of malnutrition among children and not a strategy to treat malnutrition among vulnerable populations such as internally displaced children. Hence, more intensive methods which are recommended for conflict-affected people such as supplementary feeding with ready-to-use therapeutic food and standard ready-to-use supplementary food could be used to treat malnutrition among these children.

Although the parents in the experimental group were encouraged to give food to their children three or more times a day, this had no significant impact on the children’s number of meal times per day. This is probably because the parents lacked the required resources to implement what they were taught.

Unlike most food-based behavioural interventions which focus only on increasing consumption of a single nutrient or a single food^(52,53)^, during this intervention, the mothers/caregivers were encouraged to give their children a wide variety of foods. Encouraging the consumption of a wide variety of food is particularly crucial when dealing with vulnerable populations and where there is limited information on specific nutrient deficiencies. When a whole diet approach is used, each participant in the programme is more likely to choose a health-seeking behaviour that they can easily adopt or a type of nutrient-rich food that they have access to and are willing to increase its consumption. The improvement in the frequency of consumption of fruits reported among the children in the test group is probably because fruits and vegetables are more affordable than the other food groups (animal products, legumes, and nuts) whose regular consumption was encouraged.

It is a well-known fact that dietary diversity is a major challenge for people who are poor in under developed countries, particularly in Africa. The diet of many people comprises mainly monotonous carbohydrate staples, with little or no animal products and few fresh fruits and vegetables. Optimal nutrient intake is limited by a dependence on plant-based staples like maize, cassava, and rice, as well as low-cost fats and sugar^(55,56)^. The same trend was observed in the current study.

Although lack of knowledge is one of the main reasons for low diet diversity among populations^(25)^, the results of this study portray contrary information as nutrition education did not increase the children’s DDS even though it resulted in significant improvement in nutrition knowledge of the mothers. The financial status of the mothers/caregivers of the children and prolonged food shortages are likely contributed to the negative results from the dietary practices of the children. However, a future study should be conducted to investigate the factors that are associated with the dietary practices of these children.

The food selection practices of populations are often determined by several factors. Societal factors such as cultural practices influence the type of foods eaten by a population. Also, peer pressure and family members usually make it more difficult for individuals to change their food choices and feeding habits. Generally, caregivers prefer to feed their children foods that are acceptable to their culture and are believed to have adequate nutrients for their children. One of the aims of the current study was to increase the mothers/caregivers’ knowledge of the nutritional value of native foods such that they could intentionally select a variety of foods more frequently and increase their dietary diversity. Consumption of a wide variety of foods has been associated with elevated consumption of micronutrients and hence, micronutrient adequacy. However, no significant change was recorded in the DDSs of both groups after the intervention. This was attributed to the fact that the monthly household income level of most of the mothers/caregivers (≥ 60%) was very low (less than 50,000 Francs CFA). During discussions with the mothers/caregivers in the nutrition education sessions, most of them said they like to put the lessons that they were taught into practice but were limited by the unavailability of the required foods and finances. This suggestion was confirmed by Mcnaughton^(57)^ who reported that nutrition education is only effective in mothers to change their dietary practices when income is not the limiting factor. Therefore, it has been recommended that the nutritional status of people in the poorest communities can only be effectively improved by improving their access to food^(58)^. Hence, there is a necessity to increase access to nutrient-dense foods such as eggs, fish, liver, beef, and poultry since these foods are the main sources of iron and vitamin A, which are essential nutrients for growth, immunity, and intellectual development of children^(59)^

Contrarily to the results of this study, the DDSs of rural preschool-aged children in the Ilu Abba Bor zone of southwest Ethiopia and children less than 24 months suffering from ID anaemia in rural Cameroon whose mothers participated in a nutrition education intervention increased significantly at end-line^(25,27)^. These children were not displaced and probably food availability was not a problem to them. Therefore improved knowledge resulted in improved dietary practices.

The education of mothers/caregivers of internally displaced children in the West and Littoral Regions of Cameroon resulted in insignificant improvement in the dietary diversity of the children. These results are in concordance with the findings of Mananga et al.^(27)^ who noted no significant improvement in the dietary diversity of children in rural Cameroon after a nutrition education intervention on their mothers. Correspondingly, the nutrition education of mothers of internally displaced children in Somalia did not affect the dietary diversity of their children^(45)^. Taking into consideration the multiple benefits for the mother and child from appropriate dietary practices, investigations should be done to seek alternative strategies to promote dietary diversity among internally displaced children.

Although the caregivers had significant improvement in their nutrition knowledge, the poor living conditions of internally displaced children in the West and Littoral Regions coupled with their limited family income can make it very challenging for the caregivers to access sufficient food resources or make healthy dietary decisions for their children. Other factors that may impede the caregivers from making significant behavioural improvements could be related to their emotional and mental health status. School-based interventions done within similar settings recount that children who flee from war usually experience a high prevalence of depression, worry, and terror^(60)^.

Copious evidence from similar previous studies ascertains that nutrition education may improve the knowledge of caregivers regarding healthy dietary practices and consequently nutritional status^(24–27)^. However, altering behaviours is a very complex process that might be affected by many economic, sociocultural, and environmental factors^(14,20)^. It is necessary to consider the unique population in this study and the harsh living conditions of the participants before and during the intervention as other factors that can also influence the results of the study.

The intervention was very effective in improving the health-seeking practices of the children. This could be because minimal finances were required to improve these practices. However, since optimal results (hygienic practices) were obtained after both the children and their mothers/caregivers were taught, it can be suggested that lessons on nutrition education should be taught both to the children and their mothers/caregivers to make the intervention more effective.

Apart from immunisation, the health-seeking practices of the children in both groups were sub-optimal as not up to half of them had any good health-seeking practice at baseline. The high prevalence of unhealthy practices noticed among the children at baseline might be due to insufficient knowledge, lack of emphasis, or negligence. This is because after much emphasis was placed on the significance of implementing these practices and the consequences of not practicing them, a good number of children engaged in these practices.

Nutrition and health education significantly improved all the health-promoting practices of the children in the experimental group (*p* < 0.05) except immunisation. This is in concordance with the results of Li et al.^(26)^ who also reported significant improvement in the health-seeking behaviours of migrant workers in China after an intervention programme of nutrition education was implemented. An improvement in the health-seeking practices of the children in the experimental group at the end-line indicated that the children and their mothers/caregivers and their mothers/caregivers could identify and implement desirable behaviours that would improve their hygiene and sanitation and consequently their nutritional status.

Even though the prevalence of morbidity among the children in the two groups at baseline was high, nutrition education did not affect any of the illnesses identified. This therefore signifies that the causes of morbidity among the children could be multi-factorial and could not be addressed merely by nutrition education.

The proportion of caregivers who had inadequate nutrition knowledge in both the test and control groups at the study baseline was similar and very high. This implies that most of the caregivers have inadequate knowledge of nutrition. Hence, there is a need to improve their nutrition knowledge by integrating a nutrition education programme with other interventions to prevent and treat malnutrition among these children.

At the end of the intervention, a significantly higher mean score was documented in the experimental group while the mothers/caregivers in the control group maintained their mean score. This data implies that the post-test scores of the group increased as an outcome of the nutrition education intervention. This finding is in line with a study conducted in rural Cameroon which found a substantial increase in mothers’ nutrition knowledge at the end of a nutrition education intervention programme^(27)^. Furthermore, a study conducted in Iran on the assessment of the impact of nutrition education on growth indices of Iranian nomadic children revealed a statistically significant increase in the maternal score in nutritional knowledge at the end-line assessment. The authors concluded that nutrition education is an effective tool in increasing maternal level of nutritional knowledge^(28)^.

A higher mean score on the test of knowledge denoted that the mothers/caregivers had an increased knowledge of adequate dietary practices, the nutrient content of some foods, good cooking practices, the importance of eating a wide variety of food, and the causes of nutritional deficiencies amongst others. Since nutrition education and counselling significantly augmented nutrition knowledge but not dietary practices and nutritional status, this study proves that the prevailing malnutrition amongst these children might not have been caused by a lack of nutrition knowledge only but largely by the inability to obtain the food that contains the required nutrients for the children.

One of the strengths of this study is that the mothers or caregivers were taught one-on-one mostly through phone calls. Therefore, it was ensured that during each teaching session, all the caregivers received and understood the lessons taught perfectly well. Also, they were able to express themselves and ask even questions about their private life without fear or shame. Additionally, the mothers or caregivers were given specific nutritional counselling with emphasis on how to meet the nutritional needs of their children identified during the baseline assessment.

### Conclusion

This study showed that most dietary practices of the children were sub-optimal even after the parents were taught appropriate dietary practices. The nutrition education intervention programme did not have any significant effect on the anthropometric status and nutrient status of the children but had significant beneficial outcomes for all the health-seeking practices of the children except immunisation. Nutrition education significantly reduced the prevalence of clinical signs of malnutrition studied such as Bitot’s spot and pallor at the end of the intervention. Among the dietary practices, only the children’s frequency of eating fruits and vegetables increased significantly. A significant increase in the mother/caregiver’s level of nutrition knowledge was noticed after the nutrition education intervention. This study demonstrates that nutrition education alone was not effective in improving the nutritional status of the children and should be implemented together with other food-based nutrition interventions to improve the nutritional status of internally displaced schoolchildren in the West and Littoral Regions of Cameroon.
